# The effects of killer cell immunoglobulin-like receptor (KIR) genes on susceptibility to HIV-1 infection in the Polish population

**DOI:** 10.1007/s00251-016-0906-1

**Published:** 2016-02-18

**Authors:** Katarzyna Zwolińska, Olga Błachowicz, Tomasz Tomczyk, Brygida Knysz, Jacek Gąsiorowski, Małgorzata Zalewska, Beata U. Orzechowska, Marta Sochocka, Egbert Piasecki

**Affiliations:** Laboratory of Virology, Ludwik Hirszfeld Institute of Immunology and Experimental Therapy, Polish Academy of Sciences, Weigla 12, 53-114 Wroclaw, Poland; Department of Infectious Diseases, Liver Diseases and Acquired Immune Deficiencies, Wroclaw Medical University, Wroclaw, Poland

**Keywords:** Susceptibility to HIV infection, Killer cell immunoglobulin-like receptors (KIR), Chemokine receptors, Host genetics

## Abstract

Killer cell immunoglobulin-like receptors (KIR) are the most polymorphic receptors of natural killer (NK) cells. Their activity diversifies the functions of NK cells in the antiviral immune response, so the presence of certain KIR may affect transmission of HIV-1. The aim of the study was to evaluate the influence of *KIR* genes on the susceptibility to HIV-1 infection in the Polish population depending on the route of exposure. We determined the frequencies of activating (*2DS1*, *2DS2*, *2DS3*, *2DS4f*, *2DS4del*, *2DS5*, *3DS1*) and inhibitory (*2DL1*, *2DL2*, *2DL3*, *2DL5*, *3DL1*) *KIRs* in HIV-1-positive patients (*n* = 459), individuals exposed to HIV-1 but uninfected (EU, *n* = 118) and in uninfected, healthy blood donors (BD, *n* = 98). Analysis was performed using stepwise logistic regression. Apart from *KIRs*, *CCR5-∆32*, and *CCR2-64I*, alleles were also analyzed, as we knew or suspected that these features could affect susceptibility to HIV infection. The regression confirmed the protective effect of *CCR5-∆32* (OR = 0.25, *p* = 0.006) and *CCR2-64I* (OR = 0.59, *p* = 0.032) against HIV infection. Among *KIR* genes, *2DL3* was found to be a protective factor (OR = 0.30, *p* = 0.015). A similar effect was seen for *3DS1* but only in intravenous drug users (IDUs) (OR = 0.30, *p* = 0.019), not in sexually exposed people. *2DL5* was found to be a factor facilitating HIV infection (OR = 2.13, *p* = 0.013). A similar effect was observed for *2DL2* but only in females (OR = 2.15, *p* = 0.040), and *2DS1* in IDUs (OR = 3.03, *p* = 0.022). Our results suggest a beneficial role of *KIR3DS1* and *2DL3* supporting resistance to HIV infection and a harmful effect of *2DS1*, *2DL5*, and *2DL2* genes promoting HIV acquisition.

## Introduction

The importance of host genetic factors in HIV infection has been known for the last 20 years, from the role of the mutations in genes encoding chemokines and their receptors, to specific microRNA interfering with the virus replication cycle (Arenzana-Seisdedos and Parmentier 2006; Farberov et al. 2015; Lama and Planelles 2007; O’Brien and Moore 2000; Reynoso et al. 2014; Zwolińska 2009). The nature and mode of action of these factors are very diverse. It includes the prevention of HIV entry into the cells, as it is observed for *CCR5-∆32* mutation (O’Brien and Moore 2000; Zwolińska et al. 2013a), the modulation of the immune response against virus, as it is shown in case of HLA and killer cell immunoglobulin-like receptors (KIR) (Jennes et al. 2006, 2013; Martin et al. 2002), and finally the specific epigenetic mechanisms of microRNA (Farberov et al. 2015; Reynoso et al. 2014). The role of genetic features in HIV infection is not always clear, and sometimes the results of research are ambiguous or contradictory. This applies to the mutation in chemokine receptors used as HIV co-receptors, e.g., *CCR2-64I* (Ding et al. 2011; Zapata et al. 2013), human endogenous retroviruses (Bhardwaj et al. 2014; van der Kuyl 2012; Zwolińska 2006; Zwolińska et al. 2013b), and genes encoding KIR, especially in the context of susceptibility to the virus (Hong et al. 2013; Paximadis et al. 2011; Tallon et al. 2014; Tiemessen et al. 2011).

KIR are very polymorphic structures on natural killer (NK) cells which are able to trigger their function due to passing activating or inhibitory signals. Such regulation affects both innate and adaptive immunity, particularly the antiviral response. To date, 16 *KIR* genes, 601 alleles, more than 50 haplotypes, and 398 *KIR* genotypes have been identified (González-Galarza et al. 2015; Takeshita et al. 2013). Among them, there are seven genes encoding receptors transmitting activating signals through the short cytoplasmic tail (*KIR2DS1*, *2DS2*, *2DS3*, *2DS4*, *2DS5A*, *2DS5B*, and *3DS1*), and eight genes encoding inhibitory receptors with long cytoplasmic tails (*KIR2DL1*, *2DL2*, *2DL3*, *2DL5A*, *2DL5B*, *3DL1*, *3DL2*, and *3DL3*). *KIR2DL4* may activate or inhibit NK cell activity (Faure and Long 2002; González-Galarza et al. 2015). Framework genes *KIR3DL3*, *2DL4*, and *3DL2* and pseudogene *3DP1* are present in almost all human genomes, with rare exceptions (Niepiekło-Miniewska et al. 2014). *KIR* genes are located in 15 different loci on chromosome 19q13.4, and some of them are allelic variants, *KIR3DL1*/*S1*, *2DL2/2DL3*, and *2DS3*/*2DS5* (Jiang et al. 2012; Martin and Carrington 2013). *KIR* haplotypes can possess from 7 to 12 genes and may be divided into two main haplotype groups A and B on the basis of the presence of specific genes in centromeric and telomeric regions of the cluster. Centromeric A (cenA) haplotype is characterized by the presence of *KIR2DL3* and the absence of *2DL2* and *2DS2*. The last two are determinants of centromeric B (cenB) haplotype. Telomeric A (telA) haplotype is defined by the presence of *3DL1* and *2DS4* (both, full length or form with 22-bp deletion, *2DS4del*) and the absence of *2DS1. 2DS1* and *3DS1* are typified of telomeric B (telB) haplotype, where there is neither *2DS4* nor *3DL1* (Jiang et al. 2012). Some *KIR* alleles of one gene may occupy different loci in centromeric or telomeric regions (e.g., *2DS3/2DS5*, *2DL5*) (Jiang et al. 2012). Haplotype A (cenA/telA) usually contains framework genes plus *KIR2DL1*, *2DL3*, *2DS4*, and *3DL1* (Martin et al. 2000, 2004; Takeshita et al. 2013), and therefore activating signal transmission by the KIR is limited, especially when *KIR2DS4* has a 22-bp deletion (*2DS4del*) (Graef et al. 2009). Haplotype B (cenA/telB; cenB/telA; cenB/telB) is more variable and has more combinations of activating *KIR* (*2DS1*, *2DS2*, *2DS3*, *2DS5*, and *3DS1*) (Takeshita et al. 2013; Wilson et al. 2000). Triggering activity of NK cells is associated with their receptors’ variability and expression and also with the presence of appropriate ligands, which are human leukocyte antigen (HLA) class I molecules for killer immunoglobulin-like receptors (Lanier 1998; Long and Rajagopalan 2000; Carrillo-Bustamante et al. 2015).

Literature reports tend to focus on the contribution of *KIRs* to AIDS progression rather than to susceptibility to HIV infection. Most of the researchers emphasize the role of *KIR3DS1* and *3DL1* in AIDS development, in the presence or absence of *HLA-B Bw4-80I* ligands (Gaudieri et al. 2005; Jiang et al. 2013; Martin et al. 2002, 2007). *KIR3DS1* was indicated as a protective factor, in the context of resistance to HIV during different ways of exposure (Boulet et al. 2008; Chavan et al. 2014; Habegger de Sorrentino et al. 2013; Tallon et al. 2014). Among *KIR* genes involved in susceptibility to HIV, *KIR2DS4f* was mentioned as an agent associated with accelerated HIV transmission to cohabiting seronegative partners (Merino et al. 2011) and higher risk of intrapartum transmission to children possessing *KIR2DS4del* (Hong et al. 2013). *KIR2DL3* with *HLA-C1/C2* combination was elevated in mothers transmitting HIV to their children during delivery and was lower in infected children (Paximadis et al. 2011). Some reports have indicated that HIV transmission was influenced by the compatibility of sexual partners for *KIRs* and *HLA*. Incompatibility between them may oblige NK cells from the exposed partner to rejection of incoming cells from the HIV-positive partner (Jennes et al. 2013). Also, presence of *KIRs* without their ligand, e.g., *2DL2/2DL3* without *HLA-C1* and *3DL1* without *HLA-Bw4*, may inhibit HIV infection, as was observed in the exposed to HIV but uninfected female sex workers in Côte d’Ivoire (Jennes et al. 2006). Furthermore, it seems possible that people with *KIR* genotypes with more activating genes (BB, AB) and without ligands for inhibitory *KIRs* may be better protected against HIV because of the effective NK activation (Jennes et al. 2006, 2011). There has not been enough research to uncover the effects of all *KIR* genes on HIV infection.

The main aim of the study was to evaluate the influence of genes encoding *KIR* on susceptibility to HIV-1 infection via the sexual route or intravenous drug injections. We determined the frequency of activating *KIR2DS1, 2DS2*, *2DS3*, *2DS4f*, *2DS4del*, *2DS5*, and *3DS1* and inhibitory *KIR2DL1*, *2DL2*, *2DL3*, *2DL5*, and *3DL1* in the groups of exposed to HIV and HIV-positive (HIV+) or uninfected (EU) people on the background of the blood donors from Lower Silesia region in Poland. During the preparation of the regression model for describing the impact of *KIRs* on HIV infection, we could not omit other well-known genetic factors (*CCR5-Δ32*, and *CCR2-64I*); thus, they were included in the analysis. To our knowledge, this is the first study in Poland describing the contribution of *KIR* genes to susceptibility to HIV infection.

## Patients and methods

### Patients

We included three groups of subjects in our study. The first one (BD) consisted of 98 healthy, HIV-, HCV-, and HBV-negative age-matched blood donors (40 women, 58 men) from the Lower Silesia in Poland. They were used as a control of *KIR* gene frequencies in the studied population. The other two groups comprised persons exposed to HIV but uninfected (EU, *n* = 118) or infected with the virus (HIV+, *n* = 459). The exposure to HIV in each person in EU was long-lasting and repeatable, and seronegativity was confirmed by standard laboratory tests. Of them, 71.2 % were intravenous drug users (EU-IDU; *n* = 84) with on average 13.7 years history of addiction (1.5 to 36 years); 89.3 % of them were HCV positive. Sexually exposed, long-lasting partners of HIV-infected people (EU-SE; *n* = 34) were also included in the EU group. Similar subgroups were distinguished among HIV-positive patients of the Department of Infectious Diseases, Liver Diseases, and Acquired Immune Deficiencies (Wroclaw Medical University, Poland). We included in the HIV+ cohort 359 drug addicts (HIV-IDU) and 100 sexually exposed people (HIV-SE). These people were HIV positive and were selected on the basis of a confirmed laboratory diagnosis. Both EU-IDU and HIV-IDU were also patients of Monitoring Laboratory for Infections Among Drug Users in Wroclaw, Poland. The detailed characteristics of HIV+ and EU groups are given in Table 1. All procedures performed in studies involving human participants were in accordance with the ethical standards of the institutional and/or national research committee and with the 1964 Helsinki declaration and its later amendments or comparable ethical standards. Informed consent was obtained from all individual participants included in the study. The study was approved by the Commission of Bioethics at Wroclaw Medical University (number of permission KB-182/2005).

**Table 1:** Baseline characteristics of the compared groups of people exposed to HIV, infected (HIV+), or seronegative (EU)

Group		HIV+, *n* = 459					EU, *n* = 118				
Feature		Median	Sn	Q1	Q3	CI 95 %	Median	Sn	Q1	Q3	CI 95 %
Age		29	6.1	25	36	28.4; 29.6	29	5.8	26	34	28.0; 31.0
Feature		*n*	%	CI 95 %			*n*	%	CI 95 %		
Gender	Women	153	33.3	29.0; 37.6			39	33.1	24.6; 41.5		
	Men	306	66.7	62.4; 71.0			79	66.9	58.5; 75.4		
Sexual exposure (SE)	Yes	100	21.8	18.0; 25.6			34	28.8	20.6; 37.0		
	No	359	78.2				84	71.2			
Heterosexual exposure (HT)	Yes	61	13.3	10.2; 16.4			32	27.1	19.1; 35.1		
	No	398	86.7				86	72.9			
Homosexual exposure (HO)	Yes	35	7.6	5.2; 10.1			2	1.7	0.0; 4.0		
	No	424	92.4				116	98.3			
Homo/heterosexual exposure (HO/HT)	Yes	4	0.9	0.0; 1.7			0	0.0	0.0; 0.0		
	No	455	99.1				118	100.0			
Intravenous drug users (IDU)	Yes	359	78.2	74.4; 82.0			84	71.2	63.0; 79.4		
	No	100	21.8				34	28.8			
HCV^a^	Yes	355	77.9	74.0; 81.7			77	68.1	59.6; 76.7		
	No	101	22.1				36	31.9			

*Sn* - average dispersion, *Q1*, *Q3* - 1st and 3rd quartiles, respectively, *CI 95 %* - 95 % confidence interval

^a^There were no data for HCV status for three individuals in HIV+ and five in EU group

### Genotyping

Genomic DNA was extracted from EDTA-anticoagulated blood samples using a QIAamp DNA Blood Midi Kit (Qiagen, Hilden, Germany). *CCR5-∆32* and *CCR2-64I* alleles were determined as we described previously (Zwolińska et al. 2013a). *KIR* genotyping was performed using PCR-SSP according to our previous works (Kuśnierczyk et al. 2015; Mozer-Lisewska et al. 2015). It allowed us to detect the presence of *KIR2DS1*, *2DS2*, *2DS3*, *2DS4f*, *2DS4del*, *2DS5*, *3DS1*, *2DL1*, *2DL2*, *2DL3*, *2DL5*, and *3DL1*. We did not discriminate between centromeric and telomeric *2DL5* and *2DS3/2DS5* forms.

### Data analysis

The presence of individual *KIRs* and *KIR* genotypes (AA, Bx) was calculated as the percentage of positives among all people in the studied groups. Bx represented both AB and BB genotypes, and it was characterized by the presence of one or more of the following activating genes: *KIR2DS1*, *2DS2*, *2DS3*, *2DS5*, and *3DS1*. AA genotype was defined by the presence of *KIR2DS4* (the full version *2DS4f* or *2DS4del*) and absence of mentioned activating genes characteristic for Bx. The differences in specific *KIR* and *KIR* genotype frequencies between BD, EU, and HIV+ groups were evaluated using the *χ*^2^ test. Bonferroni correction was performed in the case of multiple comparisons.

Linkage disequilibrium (LD) as a measure of association of nonrandom alleles at two different loci was used to assess the genetic association between *KIR* gene pairs in studied groups. The strength of the association between the two genes is dependent on *D’* (LD) and *r*^2^ (relative linkage) value. Their values range from 0 to 1, where 0 means independence between alleles and 1 reflects complete association between alleles at the two loci.

The evaluation of possible relations between studied *KIRs* and susceptibility to HIV infection was performed in EU (*n* = 118) and HIV+ (*n* = 459) groups, using stepwise logistic regression in the generalized linear model scheme. The equation describing the probability of HIV acquisition during exposure [*P*(*y = 1*)] is as follows: $P\left(y=1\right)=\frac{e^{\beta_1{x}_1+{\beta}_2{x}_2+ \dots +{\beta}_n{x}_n+{\beta}_0}}{1+{e}^{\beta_1{x}_1+{\beta}_2{x}_2+ \dots +{\beta}_n{x}_n+{\beta}_0}}$, where *e* is the base of the natural logarithm, *x*_*1*_, *x*_*2*_, and *x*_*n*_ are independent variables, *n* is the number of independent variables, *β*_*1*_, *β*_*2*_, and *β*_*n*_ are regression coefficients indicating the effect size of independent variables, and *β*_0_ is the intercept. Presence of the studied feature in a given individual was encoded as *x*_*n*_ = *1* and lack of it as *x*_*n*_ = *0*. Odds ratio (OR) for, e.g., variable *x*_1_, is defined by *e*^*β*1^. We analyzed the impact of the presence of *KIR* genes in the context of nongenetic factors, such as sex and route of exposure to HIV (sexual and intravenous drug use). We also analyzed presence of *CCR5-∆32* and *CCR2-64I*, as we knew they could influence HIV infection and we wanted to rule out the possibility of misinterpretation in our results. Collinearity of the covariates was assessed with the variance inflation factor (vif), and no collinearity was denoted when vif <10.0. Akaike’s information criterion (AIC) was used as a measure of fit of the models. Variables were included in the model with *p* < 0.05. All statistical analyses were performed using the platform R-CRAN version 3.1.2 (www.r-project.org).

## Results

### Distribution of *KIRs* in the Polish population of HIV-positive and HIV-negative people

The frequencies of 12 *KIRs* were evaluated in HIV-positive and HIV-exposed but seronegative people (EU) and blood donors (BD) from Lower Silesia in Poland. We also showed the distribution of *KIR* genotypes AA (with the only one activating *KIR—2DS4*) and genotypes with the more varied *KIR* sets (Bx) (Table 2). No differences in *KIR* frequencies between HIV+ patients and BD as well as between EU and BD were detected (*p* > 0.05). However, the comparison of presence of these features in EU and HIV+ groups showed statistically significant differences between frequencies of *2DL3* (95.76 vs. 83.88 %, *p* = 0.010) and *2DL5* (34.75 vs. 53.16 %, *p* = 0.004). It may suggest their opposite role in susceptibility to HIV infection—protective against virus acquisition for *2DL3* and promoting infection for *2DL5*. There were no statistically significant differences in genotypes AA and Bx between groups (*p* > 0.05). Analysis of LD between tested *KIRs* in HIV-positive, EU, and BD is shown in Table 3. It revealed similarity between LD of some gene pairs in all groups (e.g., high LD for *KIR2DS2* and *2DL2*). It also indicated differences between groups, e.g., decreased LD for *2DS1* and *3DS1* in HIV+ and EU in comparison to background population and reduced LD for pair *2DL3* and *2DL1* in HIV+ individuals compared to EU and BD.

**Table 2:** Frequencies of killer cell immunoglobulin-like receptor genes (*KIR*) and genotypes AA and Bx in studied groups

		*2DS1*	*2DS2*	*2DS3*	*2DS4f*	*2DS4del*	*2DS5*	*3DS1*	*2DL1*	*2DL2*	*2DL3*	*2DL5*	*3DL1*	Genotypes AA	Genotypes Bx
BD, *n* = 98	*n*	34	47	24	40	84	28	34	97	46	91	43	94	33	65
	%	34.69	47.96	24.49	40.82	85.71	28.57	34.69	98.98	46.94	92.86	43.88	95.92	33.67	66.33
EU, *n* = 118	*n*	35	50	24	43	101	27	37	115	53	113	41	115	44	74
	%	29.66	42.37	20.34	36.44	85.59	22.88	31.36	97.46	44.92	95.76	34.75	97.46	37.29	62.71
HIV+, *n* = 459	*n*	191	258	156	163	383	124	172	434	257	385	244	437	141	318
	%	41.61	56.21	33.99	35.51	83.44	27.02	37.47	94.55	55.99	83.88	53.16	95.21	30.72	69.28
OR		1.69	1.75	2.02	0.96	0.85	1.25	1.31	0.45	1.56	0.23	2.13	0.52	1.34	
CI 95 %		1.09	1.16	1.24	0.63	0.48	0.77	0.85	0.13	1.04	0.09	1.40	0.15	0.88	
		2.61	2.63	3.29	1.46	1.50	2.01	2.02	1.53	2.34	0.58	3.25	1.76	2.05	
p*		0.213	0.087	0.052	1.000	1.000	1.000	1.000	1.000	0.378	0.010	0.004	1.000	0.173	

*BD* - blood donors from Lower Silesia region of Poland, *EU* - exposed, *HIV*-1-seronegative, *HIV* + - exposed to HIV and seropositive, *2DS4f* - full-length *KIR2DS4* gene, *2DS4del - KIR2DS4* with 22 base pair deletion, *OR* - odds ratio, based on the comparison of EU and HIV, *CI 95* % - 95 % confidence interval, *Bx* - represents *KIR* genotypes AB and BB

*p value after Bonferroni correction (in case of comparison of *KIRs* frequencies)

**Table 3:** Linkage disequilibrium (LD) analysis of 11 studied *KIR* genes in population background (BD, part A), HIV-positive (HIV+, lower triangle, part B), and exposed but uninfected individuals (EU, upper triangle, part B)

A												B										
*2DS1*	*2DS2*	*2DS3*	*2DS4*	*2DS5*	*3DS1*	*2DL1*	*2DL2*	*2DL3*	*2DL5*	*3DL1*		*2DS1*	*2DS2*	*2DS3*	*2DS4*	*2DS5*	*3DS1*	*2DL1*	*2DL2*	*2DL3*	*2DL5*	*3DL1*
D′	0.039	**0.362**	**−1.000**	**1.000**	**0.910**	1.000	0.002	−0.125	**0.948**	**−1.000**	***2DS1***	D′	−0.056	0.171	**−1.000**	**0.842**	**0.750**	1.000	0.015	1.000	**0.650**	**−1.000**
*r* ^2^	0.001	**0.080**	**0.080**	**0.753**	**0.828**	0.005	0.000	0.002	**0.610**	**0.080**		*r* ^2^	0.001	0.018	**0.083**	**0.499**	**0.520**	0.011	0.000	0.019	**0.334**	**0.062**
		**0.760**	−0.039	−0.032	0.039	−1.000	**1.000**	**−1.000**	**0.285**	−0.039	***2DS2***	**−1.000**		**0.711**	0.410	−0.039	0.062	**−1.000**	**0.927**	**−1.000**	**0.238**	1.000
		**0.203**	0.000	0.000	0.001	0.011	**0.960**	**0.083**	**0.069**	0.000		**0.054**		**0.175**	0.004	0.000	0.002	**0.035**	**0.776**	**0.060**	**0.041**	0.019
			**−0.669**	0.183	**0.426**	1.000	**0.764**	**−0.811**	**1.000**	**−0.669**	***2DS3***	**0.374**	−0.328		−0.372	−0.272	**0.332**	1.000	**0.773**	0.017	**0.681**	−0.163
			**0.059**	0.027	**0.111**	0.003	**0.214**	**0.156**	**0.415**	**0.059**		**0.101**	0.003		0.019	0.006	**0.062**	0.007	**0.187**	0.000	**0.222**	0.003
				**−0.650**	**−1.000**	−1.000	−0.058	0.192	**−1.000**	**1.000**	***2DS4***	−0.178	−0.295	**−0.376**		**−0.676**	**−1.000**	−1.000	−0.092	−1.000	**−1.000**	**1.000**
				**0.045**	**0.080**	0.000	0.000	0.020	**0.054**	**1.000**		0.002	0.003	**0.011**		**0.054**	**0.077**	0.001	0.000	0.002	**0.066**	**0.743**
					**0.945**	1.000	−0.087	0.000	**1.000**	**−0.650**	***2DS5***	**0.931**	**0.245**	0.059	**−0.839**		**0.892**	1.000	−0.010	1.000	**0.830**	**−1.000**
					**0.673**	0.004	0.003	0.000	**0.512**	**0.045**		**0.450**	**0.017**	0.003	**0.073**		**0.517**	0.008	0.000	0.013	**0.384**	**0.088**
						1.000	0.002	−0.125	**0.948**	**−1.000**	***3DS1***	**0.871**	**0.336**	**0.426**	**−1.000**	**0.845**		1.000	0.019	−0.126	**0.793**	**−1.000**
						0.005	0.000	0.002	**0.610**	**0.080**		**0.637**	**0.053**	**0.156**	**0.064**	**0.441**		0.012	0.000	0.002	**0.539**	**0.057**
							−1.000	**1.000**	1.000	−1.000	***2DL1***	**−0.452**	**−0.635**	**0.647**	−1.000	**−0.233**	0.253		−1.000	**1.000**	1.000	−1.000
							0.012	**0.134**	0.008	0.000		**0.017**	**0.018**	**0.012**	0.002	**0.008**	0.002		0.032	**0.590**	0.014	0.001
								**−1.000**	**0.255**	−0.058	***2DL2***	**0.298**	**0.929**	**0.854**	−0.465	**0.230**	**0.326**	**−0.636**		−0.637	0.203	0.258
								**0.087**	**0.057**	0.000		**0.050**	**0.855**	**0.295**	0.007	**0.015**	**0.050**	**0.018**		0.022	0.027	0.001
									**−0.745**	0.192	***2DL3***	−0.121	**−0.568**	**−0.284**	0.018	−0.074	−0.027	**0.571**	**−0.601**		−0.081	−1.000
									**0.055**	0.020		0.004	**0.048**	**0.030**	0.000	0.003	0.000	**0.098**	**0.055**		0.001	0.001
										**−1.000**	***2DL5***	**0.765**	**0.467**	**0.945**	**−0.874**	**0.914**	**0.888**	0.097	**0.460**	**−0.654**		**−1.000**
										**0.054**		**0.368**	**0.192**	**0.405**	**0.026**	**0.272**	**0.417**	0.001	**0.189**	**0.072**		**0.049**
											***3DL1***	**−1.000**	−0.377	**−0.518**	**0.938**	**−0.751**	**−1.000**	−1.000	−0.380	0.025	**−0.806**	
												**0.071**	0.006	**0.026**	**0.672**	**0.077**	**0.084**	0.003	0.006	0.000	**0.029**	

Values marked in bold represent genes in significant LD

*D’* - linkage disequilibrium value, *r* ^*2*^ - relative linkage disequilibrium value

### Effects of *KIR* and chemokine receptor genes on susceptibility to HIV infection

We analyzed the effects of *KIRs* (Table 2) on susceptibility to HIV infection by comparison of their distribution in EU and HIV-positive people. Logistic regression was used for evaluation of probability of HIV infection depending on *KIR* genes in case of sexual exposure or intravenous drug use. We also included in the model the presence of *CCR5-∆32* and *CCR2-64I*. They were analyzed to avoid misinterpretation of effects of *KIRs* which could be caused by chemokine receptors (O’Brien and Moore 2000; Zapata et al. 2013; Zwolińska et al. 2013a). We did not find collinearity between variables included to the model (vif < 10.0, Table 4), so we could consider their effects independently. We did not prove a link between *KIR2DS2*, *2DS3*, *2DS4f*, *2DS4del*, *2DS*5, *2DL3*, *2DL5*, and *3DL1* with susceptibility to HIV infection. The genetic factors involved in susceptibility to HIV infection are shown in Table 4. Analysis confirmed a strong protective effect of *CCR5-Δ32* (*p* = 0.006), especially in females (*p* = 0.026). *CCR2-64I* was also found to be a protective factor (*p* = 0.032). Among *KIR* genes, *2DL3* was found to be a factor reducing the risk of HIV infection (*p* = 0.015) in contrast to *2DL5* (*p* = 0.013), which was found to be an agent promoting infection. The harmful role of *2DL2* was observed in women (*p* = 0.040) and *2DS1* in case of exposure to HIV via drug injections (*p* = 0.022). *3DS1* had a protective effect during exposure to HIV among IDUs (*p* = 0.019).

**Table 4:** Role of studied genetic factors in susceptibility to HIV infection

Factors	Impact^a^	β	OR	CI 95 %		*p* value	vif
*CCR5-∆32*	↓	−1.398	0.25	0.09	0.68	0.006	3.394
*CCR2-64I*	↓	−0.531	0.59	0.36	0.96	0.032	1.040
*KIR2DL3*	↓	−1.201	0.30	0.10	0.72	0.015	1.035
*KIR2DL5*	↑	0.757	2.13	1.19	3.96	0.013	1.873
*KIR2DL2* in women^b^	↑	0.767	2.15	1.08	4.70	0.040	1.190
*KIR2DS1* in IDU^c^	↑	1.109	3.03	1.21	8.13	0.022	3.486
*KIR3DS1* in IDU^c^	↓	−1.215	0.30	0.10	0.80	0.019	4.105
*CCR5-∆32* in IDU^c^	↑	1.796	6.02	2.04	18.55	0.001	2.902
*CCR5-∆32* in women^b^	↓	−1.212	0.30	0.10	0.86	0.026	1.860
Intercept	–	2.297	–	–	–	6.88e-06	–

All data come from one regression model including all factors listed in the Table 3. There was no collinearity between the variables (vif < 10.0)

↑ - infection risk increased, ↓- infection risk reduced, *β* - coefficient in regression model, *CI 95-* % 95 % confidence interval, *OR* - odds ratio, *vif* - variance inflation factor, *IDU* - intravenous drug users

^a^Impact on susceptibility to HIV infection

^b^Compared with men

^c^Compared with individuals sexually exposed to HIV

The presence of *CCR5-∆32* mutation had the biggest effect on HIV infection occurrence, as might have been expected. This allele was present in 22.0 % of EU and 16.6 % of HIV+ people. Risk of infection in people who had this allele was about four times lower (OR = 0.25) than in people with the wild version of the *CCR5*. This relation was even stronger in women (β_*CCR5-∆32:women*_ = −1.212), resulting in more than 13 times lower risk of HIV infection among *CCR5-∆32* female carriers (e^−1.3982–1.2123^ = 0.074). However, the beneficial influence of *CCR5-∆32* was six times weaker in IDUs (OR = 6.02) than in SE people. Weaker than for *CCR5-∆32*, but still a protective effect against HIV infection was observed for *CCR2-64I*. This allele was present in 28.8 % of EU and 22.2 % of HIV+ individuals. The *CCR2-64I* reduced the risk of HIV infection by about 41 % (OR = 0.59).

Logistic regression revealed that the presence of *KIR2DL3* decreased the risk of HIV infection by about 70 % (OR = 0.30). The opposite effect was observed for *2DL5*, which increased this risk more twofold (OR = 2.13) and *2DL2*, but only in women (OR = 2.15).

A contradictory role of *KIR2DS1* and *3DS1* on susceptibility to HIV on the intravenous drug use route was found. *2DS1*-positive drug addicts had three times higher risk of HIV infection (OR = 3.03) than the negative ones had. On the other hand, they had more than three times lower risk of infection when they had the *3DS1* gene (OR = 0.30).

### Distribution of allelic versions of genes *KIR2DL2/2DL3* and *KIR3DL1/3DS1* in HIV-positive and HIV-negative people

We analyzed the distribution of two pairs of *KIR* genes segregated as alleles in HIV+, EU, and BD groups (*KIR3DL1*, *3DS1* and *KIR2DL2*, *2DL3* alleles) (Table 5). Every individual had at least one of the *3DL1* or *3DS1* alleles. One of the EU individuals (0.85 %) and 13 of HIV+ (2.83 %) had neither *2DL2* nor *2DL3*. No differences in frequencies of combinations *3DL1/3DS1*, *3DL1* or *3DS1* alone as well as *2DL2/2DL3*, *2DL2* or *2DL3* alone between HIV+ patients and BD as well as between EU and BD were detected (*p* > 0.05). *2DL2* without *2DL3* was detected in 3.39 % of the EU and in 13.29 % of the HIV+ group, which indicated the harmful role of *2DL2* in case of exposure to HIV. *2DL2* presence with absence of *2DL3* resulted in more than four times higher risk of HIV acquisition (OR = 4.37, *p* = 0.005). A protective effect of the presence of *2DL3* without *2DL2* was indicated because of overrepresentation in EU compared with HIV+ (54.24 vs. 41.18 %, respectively). Hence, it decreased the risk of HIV infection by 41 % (OR = 0.59, *p* = 0.022). *2DL2/2DL3* heterozygosity was almost equally represented in both groups (Table 5).

**Table 5:** Frequencies of allelic version of genes *KIR3DL1/3DS1* and *KIR2DL2/2DL3* in HIV-positive and HIV-negative people

Groups		*3DL1*	*3DL1/3DS1*	*3DS1*	*2DL2* ^a^	*2DL2/2DL3* ^a^	*2DL3* ^a^
BD, *n* = 98	*n*	64	30	4	7	39	52
	%	65.31	30.61	4.08	7.14	39.80	53.06
EU, *n* = 118	*n*	81	34	3	4	49	64
	%	68.65	28.81	2.54	3.39	41.52	54.24
HIV+, *n* = 459	*n*	287	150	22	61	196	189
	%	62.53	32.68	4.79	13.29	42.70	41.18
OR		0.76	1.20	1.93	4.37	1.05	0.59
CI 95 %		0.49	0.77	0.57	1.56	0.70	0.39
		1.17	1.87	6.56	12.27	1.58	0.89
*p*		0.436	0.844	0.569	0.005	1.636	0.022

*p* Value after Bonferroni correction

*BD* - blood donors from Lower Silesia region of Poland, *EU* - exposed, HIV-1-seronegative, *HIV*+ exposed to HIV and seropositive, *OR* - odds ratio, based on the comparison of EU and HIV, *CI 95* % - 95 % confidence interval

^a^One of EU individuals (0.85 %) and 13 HIV+ individuals (2.83 %) had neither *2DL2* nor *2DL3*

## Discussion

Our data highlights the impact of genes encoding KIR on the risk of HIV infection in case of sexual exposure or intravenous drug use. Presence of seven activating (*2DS1*, *2DS2*, *2DS3*, *2DS4f*, *2DS4del*, *2DS5*, *3DS1*) and five inhibitory (*2DL1*, *2DL2*, *2DL3*, *2DL5*, *3DL1*) *KIRs* was determined in the group of HIV+, EU, and in healthy BD. The frequencies of *KIR*s in the BD group did not differ significantly from those given by the Allele Frequency Net Database for the Polish population (González-Galarza et al. 2015).

The differences in some *KIR* frequencies in EU and HIV+ groups indicated their involvement in susceptibility to HIV infections, e.g., *KIR2DL3* and *2DL5* (Table 2). These differences prompted us to perform more comprehensive analysis of the impact of *KIRs* on the risk of HIV infection depending on the route of exposure. We also included in the analysis alleles *CCR5-Δ32* and *CCR2-64I*. Chemokine receptors are used as main (CCR5) or alternative (CCR2, CCR3, CXCR4, and other) co-receptors for HIV, and it is known that some of their allelic versions may impact HIV infection (Arenzana-Seisdedos and Parmentier 2006; O’Brien and Moore 2000; Zwolińska 2009).

Logistic regression performed in our study revealed some important relationships between tested genetic factors and susceptibility to HIV infection (Table 3). It was no surprise that *CCR5-Δ32* had a protective effect against HIV and decreased infection risk about fourfold. There has been a lot of research in recent years confirming restriction of HIV infection by this allele (Hütter et al. 2009; Mahajan et al. 2010; O’Brien and Moore 2000; Zapata et al. 2013; Zwolińska et al. 2013a and references therein). Our analysis showed that the effect of *CCR5-Δ32* is particularly strong in females, but its favorable impact is much lower in IDUs than in sexually exposed *CCR5-Δ32* carriers (Table 3). It may be associated with high viral load during exposure by direct intravenous injections, decreased immunity, and the poor general health condition of drug addicts. In our previous work, based partially on the same patients, we proved the beneficial role of *CCR5-Δ32* during the heterosexual route of infection (Zwolińska et al. 2013a), but no effect was observed in the case of IDUs. Most of the available data showed no effect of *CCR2-64I* mutation on susceptibility to HIV infection (Mahajan et al. 2010; O’Brien and Moore 2000; Tan et al. 2010), indicating rather its involvement in the slowing of AIDS progression (Kaslow et al. 2005; O’Brien and Moore 2000; Vieira et al. 2011; Xu et al. 2010). However, there are some reports supporting a protective role of *CCR2-64I* against vertical HIV transmission (Mabuka et al. 2009) or via sexual exposure (Zapata et al. 2013). Our present analysis showed a protective effect of *CCR2-64I* against HIV acquisition. This allele resulted in a reduction of HIV infection risk by about 41 % regardless of the way of exposure (Table 3).

It is generally known that efficient activation of NK cells, their cytolytic activity, and cytokine secretion is necessary for anti-HIV reactions (Boulet et al. 2010; Jennes et al. 2006; Martin and Carrington 2013; Tiemessen et al. 2011). That is why *KIR* genotype AA may be harmful in the context of exposure to HIV because of the limited possibility for NK cell stimulation (only one activating gene, *KIR2DS4*, often defective *2DS4del*, is present in AA genotype), whereas more diverse genotypes (BB or AB), with more activating genes or any *KIR* genotypes but with no presence of ligands for inhibitory KIRs, should provide sufficient NK cell activation and thus protection against HIV (Jennes et al. 2006, 2011). We did not prove any beneficial effect of more varied genotypes of *KIRs*, and there were no differences in genotypes AA or Bx frequencies between EU and HIV+ people (Table 2). On the other hand, O’Connell et al. suggested that strong NK cell-mediated inhibition of viral replication was not absolutely necessary for the immunological control of HIV in a group of elite suppressors (ES) long-term controlling HIV viremia. Some of their ES patients with A haplotype had stronger NK-derived HIV inhibition than patients with haplotype B (O’Connell et al. 2009). Moreover, Gaudieri et al. suggested the association between haplotype B and faster CD4+ cell count decrease leading to the progression of HIV infection towards AIDS (Gaudieri et al. 2005). It might indicate that a more diverse set of KIR on NK cells (haplotype B) makes their activity regulation more difficult.

Our results allowed us to draw some unique conclusions about specific *KIR* effects on susceptibility to HIV (Table 3). Most of literature reports have focused on *KIR3DS1* and *3DL1* and their effects on HIV infection course depending on the presence of the ligand *HLA-B Bw4-80I* (Alter et al. 2007; Gaudieri et al. 2005; Jiang et al. 2013; Martin et al. 2002, 2007). We found that the risk of HIV infection in individuals with *KIR3DS1* was more than three times lower in IDUs than in SE people. *KIR3DS1*, especially when present on both chromosomes, was indicated as a protective factor during different ways of infection (Boulet et al. 2008; Chavan et al. 2014; Habegger de Sorrentino et al. 2013; Tallon et al. 2014). Our results are particularly consistent with the work of the group of Tallon, who demonstrated that the time of seroconversion in long-term repeatedly HIV-exposed people was significantly longer in homozygotes *KIR3DS1/3DS1* than heterozygotes *KIR3DS1/3DL1*. This situation occurred only among injection drug users, not in the case of initially HIV-negative partners of serodiscordant couples (Tallon et al. 2014). Mechanisms of positive action of KIR3DS1 in anti-HIV action are still unknown. Most explanations assume the effective activation of NK cells against HIV proteins via KIR3DS1 interaction with its putative ligand HLA-B Bw4-80I. It still remains controversial because there is no evidence that HLA-B Bw4-80I, a well-known ligand of inhibitory KIR3DL1, may also serve as a functional ligand for KIR3DS1 (Martin and Carrington 2013; Martin et al. 2002, 2007; Tallon et al. 2014). Moreover, it seems that the activation of NK cells via KIR3DS1 and 3DL1 is dependent on what was presented by their peptide ligands, as it was shown by O’Connor et al. (2015).

Our findings indicated that the positive effects of *KIR3DS1* among drug addicts were diminished by the presence of *KIR2DS1. KIR2DS1*-positive IDUs had more than three times higher risk of HIV acquisition than did SE individuals. To date, there has been no report about *KIR2DS1* contribution to HIV infection. Both KIR3DS1 and 2SD1 trigger NK cell function into their activation, but it occurs after binding different ligands. KIR2DS1 binds HLA-C2 (Lys80), but with low affinity, and its high-affinity ligand is not known (Martin and Carrington 2013). It is possible that expression of KIR2DS1 on NK cells does not provide their sufficient activation against HIV. Both *2DS1* and *3DS1* genes (characteristic for telomeric B haplotype) are in almost perfect LD in our BD group (*D’* = 0.910; *r*^2^ = 0.828), what is concordant with the data for Polish population (González-Galarza et al. 2015). Is interesting to note that LD for this pair is decreased in EU and HIV+ individuals (see Table 3). *2DS1* is overrepresented in HIV+. Both groups are enriched for haplotypes with *2DS1* but without *3DS1* (5.1 % for EU and 7.0 for HIV+), and, in the EU group, there are more haplotypes with *3DS1* but without *2DS1* than in the HIV+ one (6.8 vs. 2.8 %, respectively).

Another interesting finding was associated with *KIR2DL2*, *2DL3*, and *2DL5* (Table 4). We detected the protective effect of *2DL3* against HIV acquisition and the harmful role of *2DL5* and *2DL2* but only in females. Both 2DL2 and 2DL3 receptors bind to HLA-C1 (Asn80) and transfer an inhibitory signal to NK cells, but 2DL2 has higher affinity to its ligand than 2DL3 (Frazier et al. 2013). The ligand for 2DL5 has not been identified yet (Martin and Carrington 2013). Our results are not in line with investigations by Jennes et al. (2006, 2013) and Paximadis et al. (2011). Jennes et al. (2006) reported significantly higher frequencies of *KIR2DL2* and *2DL5* in a group of HIV-exposed but seronegative (ESN) than in HIV-seropositive (SP) female sex workers. *KIR2DL2*/*2DL3* heterozygotes were more frequent in the ESN (62 %) than in the SP (25 %) individuals. The opposite situation was seen in the case of homozygous *2DL3*, which was present in 14 % of ESN and 60 % of SP individuals. Furthermore, HIV restriction was observed in ESN subjects who were carriers of inhibitory *KIR2DL2*/*2DL3* but not their ligand, *HLA-C1. KIR2DL3/2DL3* homozygotes with *HLA-C1* were characteristic for HIV-seropositive female sex workers (Jennes et al. 2006), which could be due to NK cell inhibition. Paximadis and co-workers (2011) reported an increased frequency of homozygosity of *KIR2DL3* alone and in combination with *HLA-C1/C2* in mothers transmitting HIV to their children perinatally (TR) compared with mothers who did not transmit the virus (NT). On the other hand, TR had a lower frequency of the heterozygous combination *KIR2DL2/2DL3* than NT, suggesting its protective role of *2DL2* against HIV perinatal transmission to the children. Paximadis et al. (2011) reported that *KIR2DL3* in combination with *HLA-C1* and homozygosity for *KIR2DL3* with *HLAC1/C2* were both lower in infected children (INF) compared to EU children. This corresponds with our results indicating the anti-HIV effect of *KIR2DL3*.

The impact of studied *KIR* on the susceptibility to HIV in our groups may be associated with specific *KIR* haplotypes. *KIR2DL3* is a part of centromeric haplotype A, so its protective character could be also mediated by *2DL1* from this haplotype. However, *2DL1* may be also located in centromeric B haplotypes, together with *2DL5B* and *2DL2*. Our genotyping system did not allow for discrimination between centromeric *2DL5B* and telomeric *2DL5A*, and we could not determine which version was responsible for the unbeneficial effect on susceptibility to HIV. The impact of *2DL5B* could be also associated with *2DL1* and *2DL2*, and we showed harmful action of *2DL2* in the case of a woman. High positive LD between *2DL2* and *2DS2* in HIV+ and EU (see Table 3) suggested a similar role for both these genes, but we have not proven it in the regression analysis. Contribution of *2DL1* seems to be unclear due to the possibility of its location in both centromeric A and B regions. Hilton et al. showed that *2DL1* encoded by centromeric A alleles binds his ligands HLA-C2 with greater avidity than *2DL1* on the centromeric B part (Hilton et al. 2015). If the negative effect of *2DL5* is associated with telomeric B alleles, it may be also related with *2DS1*, the harmful role of which we showed in the IDU group. However, haplotypic association with susceptibility to HIV demands further clarification.

There is no doubt that NK cells are very important in immunity and that KIR are responsible for modulation of NK cells’ anti-HIV activity. However, it is quite difficult to draw general conclusions about the impact of specific *KIRs* on susceptibility and the course of HIV infection. Such analysis is specifically difficult because of the high diversity of these genes and because of HIV variability and its defensive strategies. Certainly, specific *KIR* genes may be present in many combinations as one of the 398 KIR genotypes identified or others not discovered yet (González-Galarza et al. 2015; Takeshita et al. 2013). Furthermore, they are very polymorphic; e.g., the most polymorphic *KIR3DL1/3DS1* locus has more than 70 inhibitory and more than 15 activating alleles (Robinson et al. 2013). Some alleles may be expressed at different levels; e.g., *KIR3DL1* can be expressed at a high (allele 3DL1*h) or low level (allele 3DL1*l) or even is absent on the cell surface because of internalization in the cytoplasm (allele 3DL1*004) (Martin et al. 2007). The expression of *KIR*s on the NK surface is highly varied, and two individuals may have different KIR panels on their NK, despite having the same *KIR* gene repertoire (Gardiner 2008). Besides that, the strength of NK activation or inhibition is associated with receptor–ligand relations, and even different alleles of the same gene may bind their ligands with different avidity (Hilton et al. 2015). Moreover, the activity of NK cells is dependent not only on the balance of activating/inhibitory signaling derived from KIRs but also on other receptors, e.g., NKG2, which were also found to be an HIV restriction factor (Fogli et al. 2008). The diversity in genes encoding NK cell receptors, including in different geographic populations and complexity of NK reactions during HIV infection, is probably the reason for the inconsistent or divergent results of research in this field.

This work supports the beneficial role of activating *KIR3DS1* and inhibitory *2DL3* and the unfavorable effect of activating *2DS1*, inhibitory *2DL5*, and *2DL2* genes in resistance to HIV infection. These findings are original and unique. According to our knowledge, this study for the first time takes into consideration the impact of KIR genes on susceptibility to HIV in the Poland population. Because of this, this work has some limitations, such as the relatively small study groups and the impossibility to compare our results with other analyses in the Polish general population. Moreover, we plan to extend our research on the impact of KIR and their HLA ligand combinations as well as on high-resolution *KIR* genotyping with regard to alleles with high and low expression on the NK cell surface, which requires further investigations.

In summary:

1. *CCR5-∆32* and *CCR2-64I* alleles protected against HIV infection.
2. *KIR2DL3* was found to be a protective factor and *KIR2DL5* as a factor promoting HIV infection.
3. *KIR3DS1* decreased and *KIR2DS1* increased the risk of HIV infection during intravenous drug injection but not via sexual exposure.
4. *KIR2DL2* was found to be a factor increasing the risk of HIV infection in females.
